# MiR-148a-3p/SIRT7 axis promotes glioma progression and regulates temozolomide chemosensitivity

**DOI:** 10.1007/s11060-025-05303-7

**Published:** 2025-10-20

**Authors:** Kai Sun, Jiangting Wang, Mou Gao, Jianning Zhang, Ruxiang Xu

**Affiliations:** 1 Department of Neurosurgery, Sichuan Provincial People’s Hospital, University of Electronic Science and Technology of China, Chengdu, 611731 China; 2 Department of Anesthesiology, Xiangyang Central Hospital, Affiliated Hospital of Hubei University of Arts and Science, Xiangyang, 441000 China; 3 Department of Neurosurgery, The First Medical Centre, Chinese PLA General Hospital, Beijing, 100853 China

**Keywords:** Glioma, MiRNA-148a-3p, SIRT7, Temozolomide

## Abstract

**Purpose:**

Glioma is a highly malignant primary neoplasm of the central nervous system. Temozolomide (TMZ) is the first-line chemotherapeutic drug for glioma, commonly employed in conjunction with radiotherapy to improve overall patient outcomes, but the resistance significantly limits its efficacy. Identifying regulatory molecular targets that influence glioma progression and TMZ sensitivity is critical improve treatment outcomes. Even though SIRT7 has been implicated in tumorigenesis, the regulatory mechanisms of SIRT7 and the role of its upstream microRNAs in glioma progression remain unclear.

**Methods:**

We constructed SIRT7 knockdown and overexpression in glioma cells lines, and detected the tumor phenotypes. Moreover, both in - vitro and in - vivo experiments were carried out to assess the influence of SIRT7 expression levels on the treatment effectiveness of temozolomide (TMZ) in glioma.

**Results:**

Our research indicated that SIRT7 is significantly over - expressed in tumor specimens obtained from glioma patients. This over - expression is associated with the tumor stage and a unfavorable prognosis. In addition, reducing the expression of SIRT7 can effectively impede the advancement of glioma cells. It has been confirmed that SIRT7 serves as a downstream target of miR-148a-3p. When there is an upregulation of miR-148a-3p, it suppresses the proliferation of glioma cells. Moreover, it causes tumor cells to become arrested in the G1 phase and stimulates cell death via apoptosis. Interestingly, SIRT7 knockdown enhanced TMZ-induced cytotoxicity in vitro and potentiated TMZ antitumor effects in glioblastoma xenografts.

**Conclusions:**

The aforementioned results suggested the miR-148a-3p/SIRT7 axis drives glioma progression and modulates TMZ sensitivity, and targeting this axis may represent a promising therapeutic approach to overcome TMZ resistance in glioma.

## Introduction

Glioma are highly malignant primary neoplasm of the central nervous system [1]. Epidemiological studies indicate that the yearly occurrence rate of glioma lies between 5 and 8 cases for every 100,000 people. This specific type of tumor accounts for roughly 40% of all brain tumors and about 80% of all cancerous brain tumors [2, 3]. Temozolomide (TMZ) is considered the standard primary chemotherapy medication for treating glioma. It is frequently combined with radiotherapy to enhance the overall prognosis for patients. However, the development of resistance notably diminishes its effectiveness [4–6]. Understanding the molecular mechanisms underlying glioma progression and TMZ resistance is of vital importance.

Sirtuins are a family of deacetylases that ubiquitously expressed in cells. They have seven subtypes, designated SIRT1 through SIRT7 [7, 8]. SIRT7 is enriched in the nucleolus and promotes the transcription of RNA polymerase I-dependent ribosomal RNA genes [9, 10]. It is associated with cell carcinogenicity and proliferation in melanoma [11] and colon cancer [12]. However, its role in glioma remains largely unexplored. Moreover, the upstream regulatory mechanisms that control SIRT7 expression in glioma are not well understood.

MicroRNAs (MiRNAs), which are brief non - coding RNA transcripts, participate in post - transcriptional gene regulation. They achieve this by specifically binding via hybridization to the 3’ untranslated region of target mRNAs [13]. In glioma, it is frequently observed that the regulation of miRNAs is irregular. This irregular regulation is tightly associated with tumor growth, invasion, and drug resistance. Significantly, miR-148a-3p is being more and more regarded as an anti - cancer miRNA. It acts as a crucial regulatory element in the development of various types of neoplastic disorders [14–17]. A bioinformatics study based on a glioma prognostic related miRNA risk model described seven survival-related miRNAs including miR-148a-3p [18]. Another study compared the tumor miRNA profile of glioblastoma patients with long and short survival and associated miR-148a-3p with survival [19]. Taken together, the current data underscore miR-148a-3p as a prospective molecular target in glioma.

In this current study, we carried out a thorough analysis to understand the workings of the miR-148a-3p/SIRT7 molecular axis in glioma. Our focus was on how this axis regulates the proliferation of glioma cells, induces apoptosis, and causes cell cycle arrest. Moreover, we comprehensively gauged its impact on the sensitivity of glioma cells to temozolomide (TMZ) chemotherapy. We employed both in vitro cell - based tests and in vivo xenograft mouse models for a systematic evaluation. Our findings provide a novel regulatory mechanism for glioma progression and a potential therapeutic strategy to overcome TMZ resistance.

## Materials and methods

### Bioinformatics analysis

In our research, the data employed was sourced from two publicly accessible databases: the Chinese Genome Glioma Atlas (CGGA) and the Cancer Genome Atlas (TCGA). The TCGA database offered RNA - sequencing data along with clinical information from 702 patients. Conversely, the CGGA database furnished RNA - sequencing data and clinical particulars from 325 patients. Our research project adhered to the Helsinki Declaration and received authorization from the ethics review panel of the Chinese PLA General Hospital. This authorization confirmed that the patients’ informed consent had been secured for both public data repositories. Subsequently, we examined the associations between the expression amounts of SIRT7 messenger ribonucleic acid (mRNA) and typical prognostic characteristics. such as the World Health Organization classification, IDH wildtype/mutant, 1p/19q coding/non-coding, MGMT methylated/un-methylated. In addition, we analyzed the relationship between patients’ clinical prognosis and SIRT7 expression.

### Tissue samples and cell culture

A total of 18 samples of primary glioma tissue and 6 samples of non - neoplastic brain tissue were procured by the neurosurgery department at the First Medical Center of the Chinese PLA General Hospital. Normal human astrocytes (NHA) and several human glioblastoma cell lines were furnished by the cell bank of the Chinese Academy of Sciences in Shanghai, China. The cell lines consisted of U87MG, U251, A172, U118MG, T98G, and LN229. Cell lines of glioblastoma were grown in Dulbecco’s Modified Eagle’s Medium that had a high level of glucose (Product No. 21013024, Gibco, USA). To this culture medium, 10% Fetal Bovine Serum (Product No. A5670701, Gibco, USA) and 1% penicillin/streptomycin (Product No. 15140122, Gibco, USA) were added. Conversely, NHA cells were incubated in astrocyte medium (Product No. #1801, ScienCell, USA). Each individual cell was maintained at a temperature of 37 degrees Celsius within a humidified incubator that had an atmosphere consisting of 5% carbon dioxide.

### Reagents and transfection

Specifically, we explored whether microRNAs may post-transcriptionally regulate SIRT7 expression, TargetScan 7.2, starBase v3.0 and miRDB were utilized to predict target mircoRNAs. The miR-148a-3p mimics, miR-148a-3p inhibitors, microRNA negative control (miR-NC), and inhibitor negative control (inhibitor-NC) were procured from OBiO Technology, a company located in Shanghai, China. Genechem, another Shanghai-based firm in China, supplied the lentivirus packages of SIRT7 short hairpin ribonucleic acid (shSIRT7), SIRT7 negative control (shNC), along with the pcDNA - SIRT7 overexpression plasmid (OE) and its corresponding negative control (OENC). Temozolomide (TMZ) was acquired from Selleck.cn (product number S1237, China). In accordance with the instructions furnished by the manufacturer, the glioblastoma cells underwent transfection using the Lipofectamine™ 3000 transfection reagent (product number L3000015, Thermo Fisher Scientific, USA). These cells were transfected with either 100 nanomoles per liter (nM) of miR-148a-3p mimics or 200 nM of miR-148a-3p inhibitors. A ratio was set where for every 0.04 nanomoles (nmol) of miRNA mimics or inhibitors, 2.5 µl (µl) of the transfection reagent was used.

### Reverse transcription-quantitative PCR (RT-qPCR) assay

According to the manufacturer’s guidelines, the TRIzol™ Reagent (15596026CN, Invitrogen, USA) was used to extract ribonucleic acid (RNA) from clinical tissue specimens and cells cultured in the laboratory. To quantify the expression level of miR-148a-3p, the TaqMan MicroRNA Assay kit (4427975, Thermo Fisher Scientific, USA) was employed. The All - in - One™ miRNA First - Strand cDNA Synthesis Kit 2.0 (Product Code QP113, produced by GeneCopoeia in China) was utilized to transform the extracted ribonucleic acid (RNA) into complementary deoxyribonucleic acid (cDNA) via reverse transcription. Subsequently, the PowerTrack™ SYBR Green Master Mix, which is intended for quantitative polymerase chain reaction (qPCR) (Product Code A46109, provided by Thermo Fisher Scientific in the USA), was applied for quantification on the Roche LightCycler480 system (Product Code 900066, made by Roche in Switzerland). Glyceraldehyde 3-phosphate dehydrogenase(GAPDH), functioning as a constitutive gene, was employed to standardize the expression of Sirtuin 7(SIRT7).The sequences of the quantitative primers for SIRT7 are presented below.The forward primer had the sequence 5’-AGCACGGCAGCGTCTATC-3’,and the reverse primer had the sequence 5’-CATGTGGGTGAGGGTTGG-3’.Regarding GAPDH, the forward primer had the sequence 5’-TCCACATGAAGTGTGACGT-3’,and the reverse primer had the sequence 5’-TACTCCTGCTTGCTGATCCAC-3’.

### Western blot assay

A mixture of RIPA extraction buffer (R0278, Sigma - Aldrich, Germany) and a protease inhibitor cocktail (P1011, Beyotime, China) was employed to lyse cells and tissues. Subsequently, the BCA protein assay kit (PC0020, Solarbio, China) was utilized to measure the protein concentrations of the test samples. A quantity of 30 micrograms of protein was subjected to separation via sodium dodecyl sulfate - polyacrylamide gel electrophoresis. Subsequently, the separated proteins were transferred onto PVDF membranes (88518, Thermo Fisher Scientific, USA). To begin with, the PVDF membranes were subjected to a blocking procedure using 5% Bovine Serum Albumin (BSA) (product number ST023, Beyotime, China). Following this, these membranes were incubated at 4 degrees Celsius overnight with primary antibodies. The specific primary antibodies employed in this experiment were those targeting SIRT7 (product number 5360, CST, USA) and GAPDH (product number 2118, CST, USA). Afterward, a secondary antibody, namely a goat - anti - rabbit antibody conjugated with horseradish peroxidase (HRP), was added to bind to the primary antibodies (7074 and 7076, CST, USA). Next, the PVDF membranes were permitted to react with Immobilon Western Chemiluminescent HRP Substrate (P90719, Millipore, USA) for a period ranging from 10 to 15 s. Ultimately, a quantitative densitometry analysis of the protein bands was performed using Quantity One Software (Bio - rad, USA).

### Luciferase reporter assay

The psiCHECK − 2 luciferase vector was engineered to incorporate segments of the wild - type SIRT7 3′ untranslated region (UTR). It is hypothesized that these segments harbor binding sites for miR-148a-3p. Additionally, the vector was built to include their mutated counterparts (Promega, Madison, WI, USA). After that, luciferase reporters for SIRT7 3’ UTR - wild type (WT), SIRT7 3’ UTR - mutant 1 (MUT1), and SIRT7 3’ UTR - mutant 2 (MUT2) were produced respectively. Next, glioblastoma cells that had been transfected with miR-148a-3p mimics and the negative control miR - negative control (NC) were co - transfected with the previously mentioned reporters. Forty - eight hours after transfection, a dual luciferase reporter assay system from Promega was utilized to measure the luciferase activities.

### Cell counting kit-8 (CCK-8) assay

The capacity of glioblastoma cells to multiply was determined using the CCK − 8 assay (C0041, Beyotime, China). Specifically, the cells were transferred into 96 - well plates. After the cells had been infected or treated, 10 µl of CCK − 8 solution was introduced into each well. Subsequently, the plates were incubated at 37 °C for a duration of 2 h. Following this, the absorbance at a wavelength of 450 nm (OD450) was measured.

### 5-ethynyl-2’-deoxyuridine (EdU) assay

The BeyoClick™ EdU − 594 cell proliferation assay kit (C0078L, Beyotime, China) was employed to measure the DNA synthesis of glioblastoma cells. One day before the experiment, glioblastoma cells were plated into 24 - well plates. Then, following the instructions provided by the product’s manufacturer, the pre - heated EdU working solution was added. After adding the solution, the cells were placed in an incubator maintained at 37 °C for 2 h to perform EdU labeling. Once the labeling was completed, the cells were fixed with a 4% paraformaldehyde solution. Subsequently, 500 µl of the Click reaction solution was added, and the cells were incubated in the dark at room temperature for 30 min. After that, 1 milliliter of the Hoechst 33,342 solution was added, and the incubation was continued in the dark at room temperature for 10 min. Eventually, the cells were examined using a fluorescence microscope (Olympus BX51, Japan).

### Mouse experiments

All experimental procedures were conducted in strict accordance with the Guidelines for the Welfare and Utilization of Laboratory Animals promulgated by the National Institutes of Health. Moreover, these procedures received authorization from the Animal Experiment Ethics Committee of the Chinese PLA General Hospital (Authorization number: 2018–066). Four groups were randomly formed using 3–5 - week - old male Balb/c nude mice. The first cohort was the control cohort, simply named “Control”. The second was the temozolomide (TMZ) cohort. The third was the shSIRT7 cohort, and the fourth was the TMZ + shSIRT7 cohort. Each of these cohorts was composed of three mice. Subsequently, 5 million U251 cells were separately injected beneath the skin of the left upper limb of each mouse. One week after the injection of cells, temozolomide (TMZ) was administered via intraperitoneal injection. The dose was established at 20 milligrams per kilogram of the body’s weight, and this therapy was conducted on a daily basis for a span of two weeks. Thirty - five days after the initial cell injection, the tumors were excised surgically and then measured for weight.

### Immunohistochemistry

The tissue specimens were immobilized with a 4% paraformaldehyde solution. Subsequently, they were encased in paraffin, and slices of suitable thickness were obtained using a microtome. At room temperature, the slices were deparaffinized using xylene and then rehydrated with absolute ethanol. Antigen retrieval was achieved by immersing the slices in a sodium citrate solution within a 98℃ hot water bath for 15 min. To avoid non - specific attachment, a goat serum blocking solution was applied at ambient conditions. The tissue specimens were exposed to a SIRT7 antibody (product code 12994–1 - AP, sourced from Proteintech, USA) and a Ki − 67 antibody (product code 27309–1 - AP, also procured from Proteintech, USA). The specimens were then subjected to an overnight incubation at 4℃ to enable the antibodies to interact with the sections. On the subsequent day, the slices were initially stored at ambient temperature for half an hour. Subsequently, the antibodies were eliminated via a washing process. Afterward, 50 µl of a Streptomyces antibiotic - peroxidase mixture was added and incubated at room temperature for 10 min. Ultimately, this mixture was washed away. Two droplets of DAB solution, totaling 100 µl, were added to each slide. After that, the slides were put under a microscope for a 10 - minute examination. Once the staining reached an ideal state, the staining process was halted, and the slides were washed. Hematoxylin was used for counter - staining, and the counter - staining lasted for 2 min. As soon as the cell nuclei turned blue, the counter - staining was ended, and the slides were washed once more. Finally, the slices were left to air - dry before being photographed.

### Statistical analysis

Each experiment was carried out independently a minimum of three times. All the data mentioned earlier was analyzed using GraphPad Prism8 software. To assess the differences statistically, the Student’s t - test, one - way ANOVA, and the Tukey post - hoc test were employed. A P - value less than 0.05 was regarded as statistically significant.

## Results

### SIRT7 was significantly upregulated in glioma tissues and cells and was correlated with glioma grade and molecular types

**Fig. 1:**
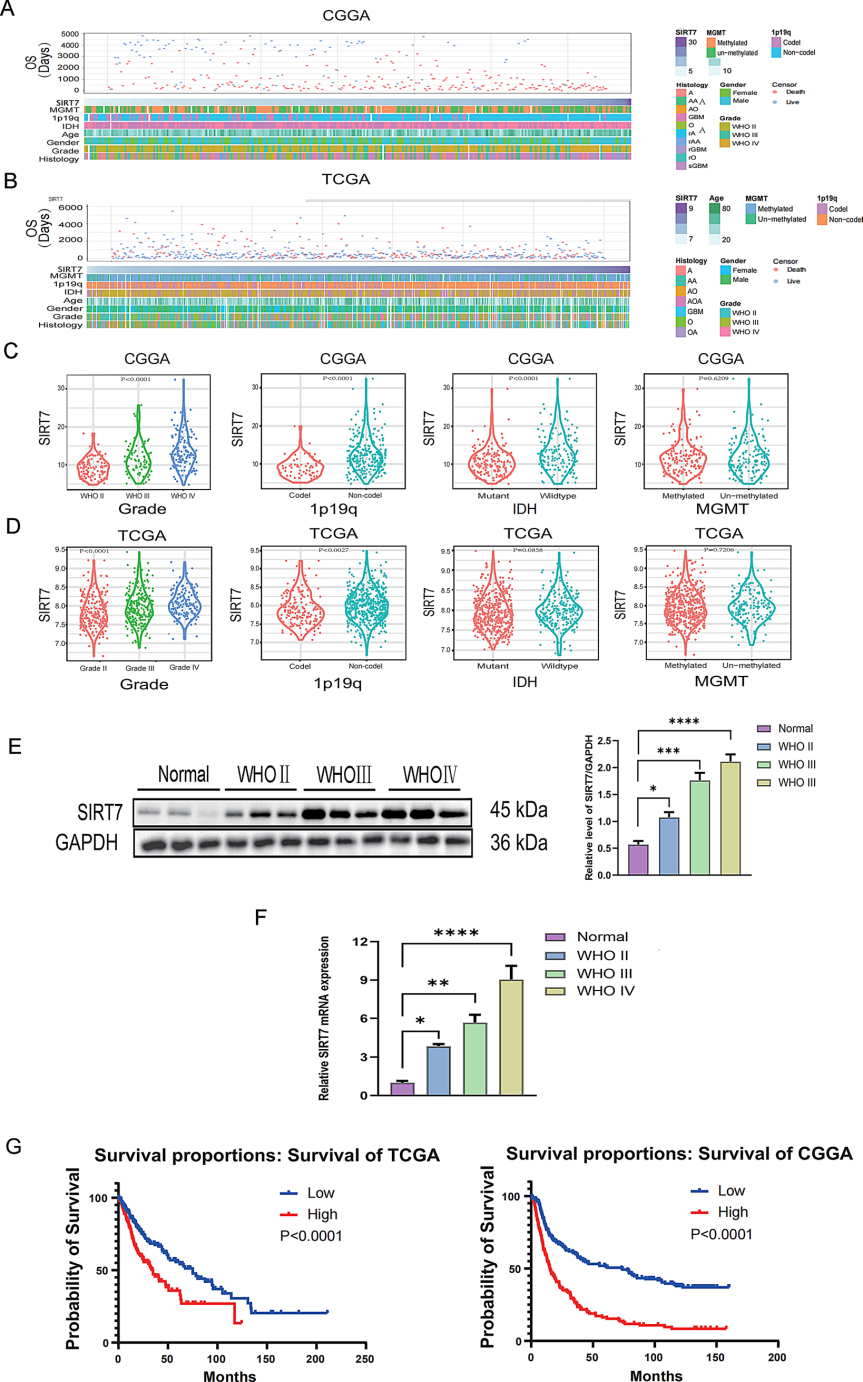
Expression of SIRT7 and survival analysis in glioma. (**A**,** B**) Comprehensive analyses of SIRT7-related clinicopathological features were performed using publicly available CGGA (**A**) and TCGA (**B**) Datasets, encompassing demographic variables, histological grades, and molecular markers of glioma patients. (**C**,** D**) Violin plots illustrated SIRT7 expression profiles in TCGA (**C**) and CGGA (**D**) Cohorts according to the 2021 WHO Classification of CNS Tumors. Statistical analyses (one-way ANOVA for multi-group comparison and unpaired t-test for binary comparison) revealed significant intergroup differences in SIRT7 expression across glioma grades. (**E**,** F**) Clinical samples (normal brain tissues, WHO grade II, WHO grade III, WHO grade IV gliomas tissues, *n* = 3) analyzed by Western blot (**E**) and RT-qPCR (**F**) showed consistent upregulation of SIRT7 at both protein and mRNA levels with increasing tumor malignancy. Expression levels were normalized to GAPDH. For the data presented in (**E** - **F**), multiple groups used one-way ANOVA test and Tukey’s post-hoc test. Significance was set at *p* < 0.05. (*p < 0.05, **p < 0.01, ***p < 0.001, ****p < 0.0001) (**G**) Survival evaluations carried out using the TCGA and CGGA datasets demonstrated that glioma patients who had lower SIRT7 expression exhibited significantly better overall survival than those with higher expression. This result was determined via Kaplan - Meier analysis (log - rank test, *p* < 0.05), and the significance levels were marked as *p < 0.05 and ****p < 0.0001

Bioinformatics analysis showed that SIRT7 expression, 1p/19q coding status, IDH mutation status, WHO grade, and histological diagnosis were asymmetrically distributed in the CGGA and TCGA datasets (Fig. 1A-B). The levels of SIRT7 were examined in patients with various glioma subtypes. The findings indicated that, within the CGGA dataset, there was a notable positive association between the expression of SIRT7 and the combined molecular - clinical characteristics. These characteristics included a high - grade classification, the IDH wild - type state, and the non - deletion of 1P/19q. (Fig. 1C). In the TCGA datasets, patients with high-grade, IDH wild-type tumors exhibited remarkably elevated SIRT7 expression. Notably, no statistically significant differences were observed between patients with 1p/19q codeleted tumors and those with non-codeleted tumors subgroups, which may be due to tumor heterogeneity (Fig. 1D). Using RT-qPCR and western blot assays, we comparatively analyzed SIRT7 expression between glioma specimens and matched adjacent non-tumoral brain tissues obtained from patients at the Chinese PLA General Hospital. According to Fig. 1E and F, the statistical results of SIRT7 protein expression in normal brain tissues vs. gliomas tissues (*F(3, 8) = 37.22, p < 0.0001*) are presented below: normal brain tissues vs. WHO grade II glioma tissues: 0.57 ± 0.11 vs. 1.08 ± 0.16 (*p* = 0.0327), normal brain tissues vs. WHO grade III glioma tissues: 0.57 ± 0.11 vs. 1.76 ± 0.24 (*p* = 0.0002), normal brain tissues vs. WHO grade IV glioma tissues: 0.57 ± 0.11 vs. 2.11 ± 0.24 (*p < 0.0001*). The statistical results of SIRT7 mRNA expression in normal brain tissues vs. gliomas tissues (*F(3, 8) = 28.66, p = 0.0001*) are presented below: normal brain tissues vs. WHO grade II glioma tissues: 1.00 ± 0.26 vs. 3.83 ± 0.30 (*p* = 0.0321), normal brain tissues vs. WHO grade III glioma tissues: 1.00 ± 0.26 vs. 5.67 ± 1.08 (*p* = 0.002), normal brain tissues vs. WHO grade IV glioma tissues: 1.00 ± 0.26 vs. 9.04 ± 1.85 (*p < 0.0001*). These results suggest that SIRT7 upregulation in gliomas may contribute to tumor progression, as evidenced by its significant overexpression in tumor tissues relative to normal controls and its positive correlation with histological grade advancement (Fig. 1E and F). In addition, we used patient information from the TCGA and CGGA databases for prognostic analysis. In the meantime, patients exhibiting low expression levels of SIRT7 had a notably more favorable clinical prognosis compared to those with high SIRT7 expression (Fig. 1G). These findings indicated that SIRT7 was mainly found in gliomas and was linked to malignancy.

### Knockdown of SIRT7 significantly suppressed glioma cell proliferation and arrested cell cycle progression, while promoting apoptotic cell death

**Fig. 2:**
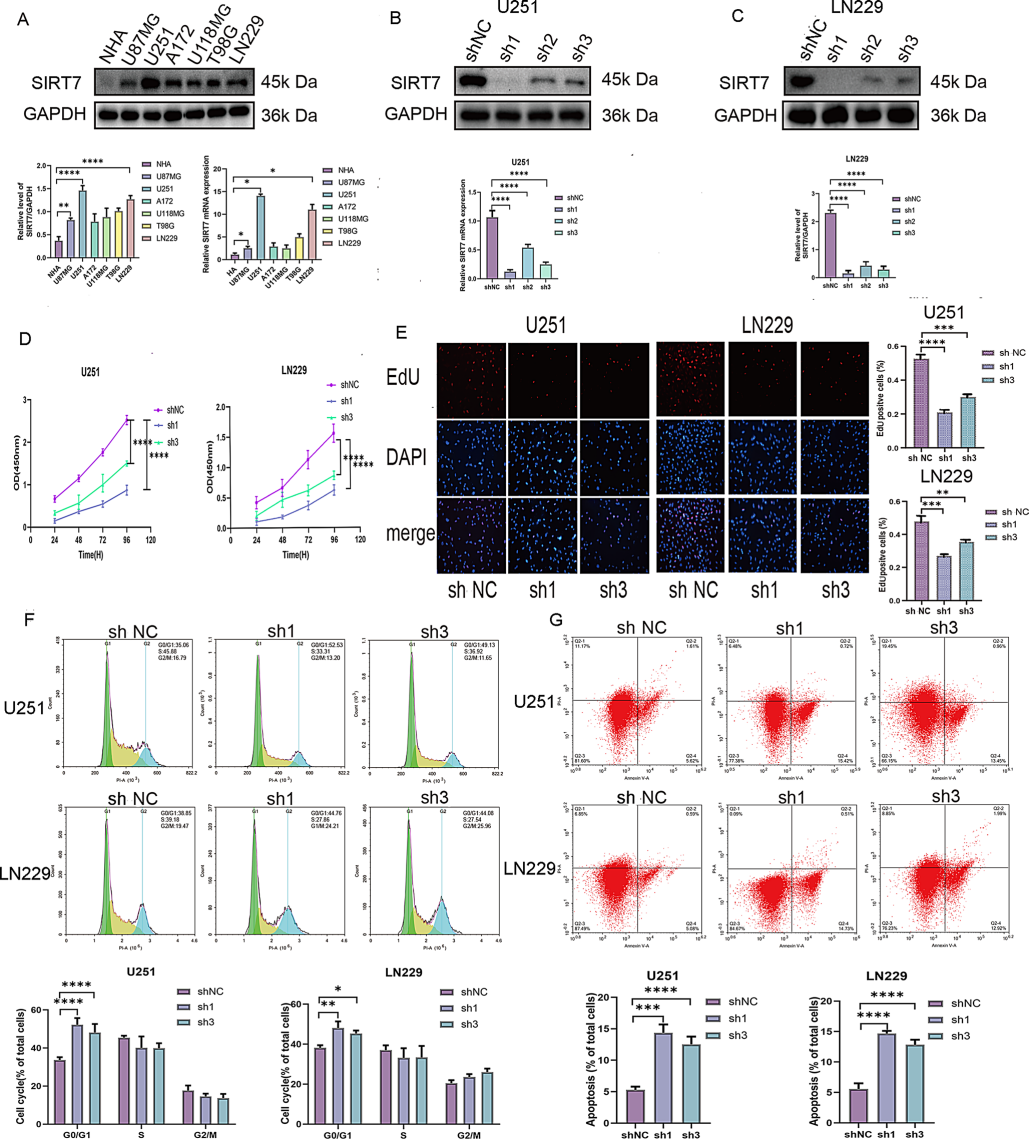
Effect of SIRT7 downregulation on the abilities of proliferation, cell cycle and apoptosis in glioma cells. (**A**) Western blotting results and RT-qPCR results of SIRT7 expression in NHA and human glioblastoma cell lines (U87MG, U251, A172, U118MG, T98G, and LN229). (**B**,** C**) The knockdown effciency of shSIRT7 was analyzed by RT-qPCR and western blot in U251 cells (**B**) and LN229 cells (**C**). SIRT7 knockdown Control group (ShNC), shSIRT7 No.1 sequence group (sh1), shSIRT7 No.2 sequence (sh2), shSIRT7 No.3 sequence (sh3). (**D**) The proliferative capacity of cells was evaluated via the CCK − 8 assay. (**E**) Images of EdU staining were obtained for U251 and LN229 cells with downregulated SIRT7 expression. (Magnification: 10×). (**F**) Flow cytometry was employed to assess the cell cycle. (**G**) Flow cytometry was also used to measure cell apoptosis. For the data presented in (**A - G**), two-group comparisons used Student’s t-test, and multiple groups used one-way ANOVA test and Tukey’s post-hoc test. Significance was set at *p* < 0.05 (*p < 0.05, **p < 0.01, ***p < 0.001, ****p < 0.0001)

Given the significant upregulation of SIRT7 in high-grade glioma and its association with poor patient prognosis, we next investigated the biological function of SIRT7 in glioblastoma cells using loss-of-function approaches. First, We evaluated SIRT7 expression levels in normal human astrocytes and glioma cells (Fig. 2A). U251 and LN229 cells with relatively high SIRT7 expression levels were infected with a shSIRT7-expressing lentivirus to knock down expression (shSIRT7), and U87MG cells with relatively low SIRT7 expression were infected with SIRT7 cDNA-expressing lentivirus to increase expression (OE). According to Fig. 2B and C, the statistical results of the effects of three distinct shRNA sequences designed by Genechem(Shanghai, China) to show that the knockdown efficiency of SIRT7 protein in U251 cells (*F(3*,* 8) = 115*,* p < 0.0001*) are presented below: shNC vs. sh1: 1.07 ± 0.11 vs. 0.12 ± 0.03 (*p < 0.0001*), the knockdown efficiency reached 90%; shNC vs. sh2: 1.07 ± 0.11 vs. 0.54 ± 0.05 (*p < 0.0001*), the knockdown efficiency was 50%; shNC vs. Sh3: 1.07 ± 0.11 vs. 0.25 ± 0.04(*p < 0.0001*), the knockdown efficiency reached 77%. The knockdown efficiency of SIRT7 protein in LN229 cells (*F(3*,* 8) = 244.8*,* p < 0.0001*) are presented below: shNC vs. sh1: 2.31 ± 0.10 vs. 0.15 ± 0.10 (*p < 0.0001*), the knockdown efficiency reached 94%; shNC vs. sh2: 2.31 ± 0.10 vs. 0.43 ± 0.14 (*p < 0.0001*), the knockdown efficiency was 80%; shNC vs. Sh3: 2.31 ± 0.10 vs. 0.29 ± 0.12 (*p < 0.0001*), the knockdown efficiency reached 88%. Given the above findings, shSIRT7 No.1 and No.3 sequences were chosen to infect U251 and LN229 cells respectively because of their role of efficiently and significantly knocking down SIRT7 expression (Fig. 2B-C). Initially, we employed the CCK − 8 and EdU assays to evaluate the effect of decreased SIRT7 expression on the proliferation of tumor cells. The findings showed that inhibiting SIRT7 (shSIRT7) hindered cell proliferation (Fig. 2D - E). Afterward, we carried out quantitative evaluations of the cell cycle progression and apoptosis rates in glioma cells. We compared two groups: a control group, in which the cells were transfected with shNC, and an experimental group, where the cells were transfected with shSIRT7. The findings showed that the cells transfected with shSIRT7 were halted in the G1 phase (Fig. 2F). Moreover, there was a notable increase in the number of apoptotic cells (Fig. 2G).

### SIRT7 overexpression in glioblastoma cells promoted proliferation and cell cycle progression and reduced apoptosis

**Fig. 3:**
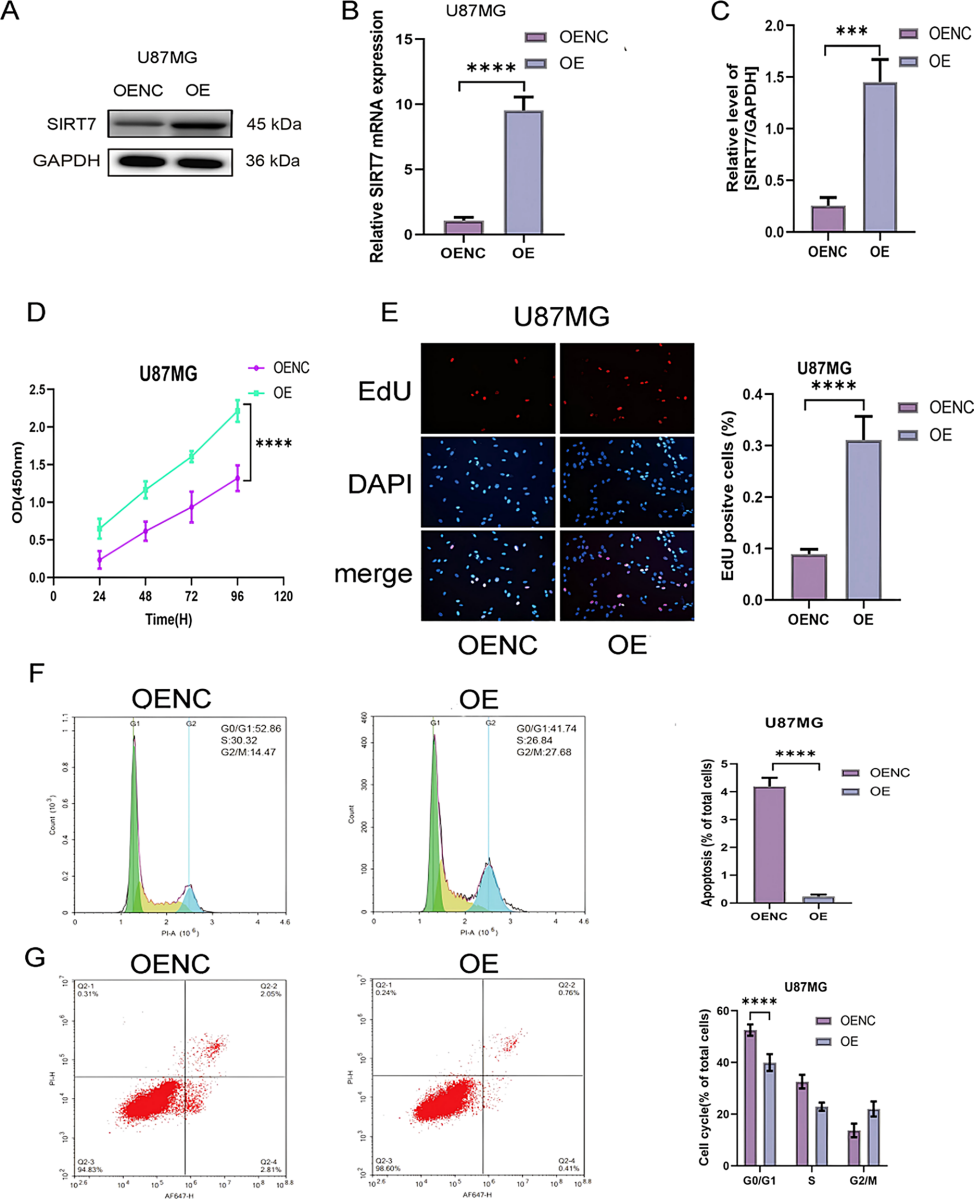
Effect of SIRT7 overexpression on the abilities of proliferation, cell cycle and apoptosis in glioma cells. (**A-C**) The overexpression effciency of cDNA SIRT7 lentivirus was analyzed by qRT-PCR and western blot in U87MG cells. SIRT7 overexpression group (OE), SIRT7 overexpression control group (OENC). Cell viability, proliferation, cell cycle, and apoptosis were assessed using CCK-8 (**D**), EdU (**E**), and flow cytometry (**F**,** G**). Statistical analysis used Student’s t-test for two groups, one-way ANOVA test and Tukey’s post-hoc test for multiple comparisons. Significance was set at *p* < 0.05 (*p < 0.05, **p < 0.01, ***p < 0.001, ****p < 0.0001)

To further dissect the functional significance of SIRT7 in glioma tumorigenesis and malignant progression, we next examined whether forced overexpression of SIRT7 could reverse the effects observed in the knockdown experiments. When U87MG cells are infected with cDNA SIRT7 lentivirus (this group is termed the OE group), there is an up - regulation of SIRT7 expression at both the mRNA and protein levels. This situation stands in contrast to that of the negative control group (OENC). (Figure 3A and C). Upon SIRT7 overexpression, CCK-8 and EdU assays showed faster proliferation in U87MG cells of the OE group versus the OENC group. (Fig. 3D-E). Flow cytometry showed that OE group showed faster cell cycle progression (Fig. 3F) and reduced apoptosis (Fig. 3G) than those in OENC group.

### SIRT7 is a validated downstream target of miR-148a-3p

**Fig. 4:**
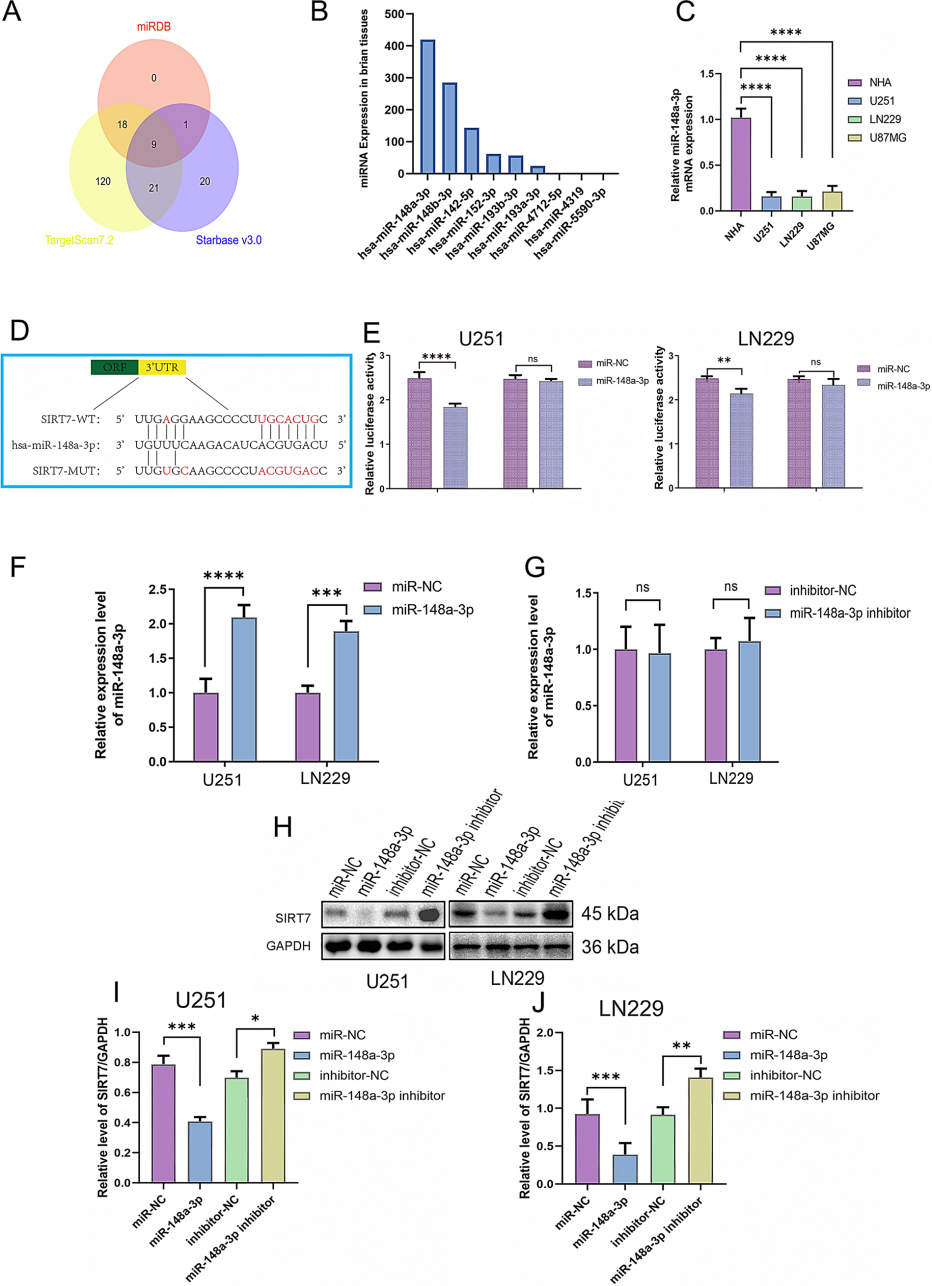
miR-148a-3p negatively regulates SIRT7 protein expression in glioma cells. (**A**) Venn diagram in the predictions among three miRNA databases. (**B**) The average expression of miRNAs in human brain tissues according to miRNA Tissue Atlas 2025 database. (**C**) The mRNA expression of miRNA-148a-3p in NHA, U251, LN229, U87MG glioblastoma cells by RT-qPCR (n = 3). (**D**) in silico analysis was carried out. This analysis pinpointed potential binding sites for miR-148a-3p within the 3’ - untranslated region (3’ - UTR) of SIRT7 mRNA, as shown in the schematic illustration. (**E**) Luciferase activity in different mimic groups is assayed by dual luciferase assay (*n* = 3). (**H-J**) Western blot analysis was performed to analyze the expression of SIRT7 protein in different groups. In these experiments, the protein levels were standardized relative to GAPDH. For the data presented in (**A - J**), two-group comparisons used Student’s t-test, and multiple groups used one-way ANOVA test and Tukey’s post-hoc test. Significance was set at *p* < 0.05 (*ns: p > 0.05*, *p < 0.05, **p < 0.01, ***p < 0.001, ****p < 0.0001)

Considering the well-documented oncogenic function of SIRT7 in driving glioma progression, we next sought to investigate its upstream regulatory mechanisms. We pooled the miRNAs predicted by the three databases(TargetScan 7.2, starBase v3.0 and miRDB) and took their intersection to generate a candidate list (miR-193a-3p, miR-142-5p, miR-148b-3p, miR-152-3p, miR-193b-3p, miR-148a-3p, miR-4712-5p, miR-4319, miR-5590-3p) (Fig. 4A). To further narrow down the target, we queried the miRNA Tissue Atlas 2025 (https://ccb-compute2.cs.uni-saarland.de/mirnatissueatlas_2025/) for the abundance of each candidate in human brain cell lines. Candidates were ranked by expression level, and the miRNA with the highest abundance was selected for downstream validation (miR-148a-3p) (Fig. 4B). Its expression was subsequently quantified in NHA and in the glioblastoma cell lines U251, LN229 and U87MG, an analysis using quantitative reverse transcription - polymerase chain reaction (qRT - PCR) revealed that the expression levels of miR-148a-3p were notably lower in the U87MG, U251, and LN229 glioma cell lines when contrasted with normal human astrocytes (NHA) (Fig. 4C). Predictions and subsequent confirmations indicated that the 3’-untranslated segment (3’-UTR) of SIRT7 messenger ribonucleic acid (mRNA) harbors a sequence that corresponds to the seed region of miR-148a-3p (Fig. 4D). Dual-luciferase reporter experiments showed that when miR-148a-3p was overproduced, it markedly reduced the relative luciferase activity of the reporter construct with the wild-type 3’-UTR of SIRT7 in the U251 and LN229 glioma cell lines. Significantly, overexpressing miR-148a-3p did not lead to substantial alterations in the luciferase activity of the mutant reporter construct for the SIRT7 3’-UTR. This outcome verified the specificity of the binding of miR-148a-3p to the wild-type seed sequence (Fig. 4E). This discovery indicates that miR-148a-3p engages with the 3’-UTR of SIRT7 at the expected binding locations, which implies that SIRT7 is indeed a downstream target of miR-148a-3p. In U251 and LN229 cells, a notable elevation in the levels of miR-148a-3p was observed after the introduction of miR-148a-3p mimics, the statistical results of SIRT7 protein expression in miR-NC vs. MiR-148a-3p(mimics) (*t = 6.973*,* df = 4*,* p = 0.0022*) in U251 cells, and (*t = 8.551*,* df = 4*,* p = 0.0010*) in LN229 cells (Fig. 4F). However, no statistical differences were observed after the introduction of miR-148a-3p inhibitors(*t = 0.1796*,* df = 4*,* p = 0.8662*) in U251 cells and (t = 0.5547, df = 4, *p* = 0.6087) in LN229 cells (Fig. 4G). Moreover, the expression of SIRT7 in U251 cell was considerably reduced in cells treated with miR-148a-3p-mimics (with miR-NC vs. miR-148a-3p: 0.7870 vs. 0.4077 (*p < 0.0001*), (*F (3*,* 8) = 69.68*,* P < 0.0001*)), and the expression of SIRT7 in U251 cell was elevated in cells treated with miR-148a-3p-inhibitors (with inhibitor-NC vs. miR148a-3p inhibitor: 0.6989 vs. 0.8901 (*p = 0.0012*)). In LN229 cells, the statistical results of miR-NC group vs. miR-148a-3p group: (*F (3*,* 8) = 24.43*,* p = 0.0002*), miR-NC vs. miR-148a-3p: 0.9238 vs.0.3886 (*p = 0.0040*), inhibitor-NC vs. miR148a-3p inhibitor: 0.9141 vs. 1.405 (*p = 0.0066*) (Fig. 4H - J). These outcomes suggest that SIRT7 is a verified downstream target of miR-148a-3p.

### miR-148a-3p regulated proliferation, cell cycle proliferation, and apoptosis in glioblastoma cells by targeting SIRT7

**Fig. 5:**
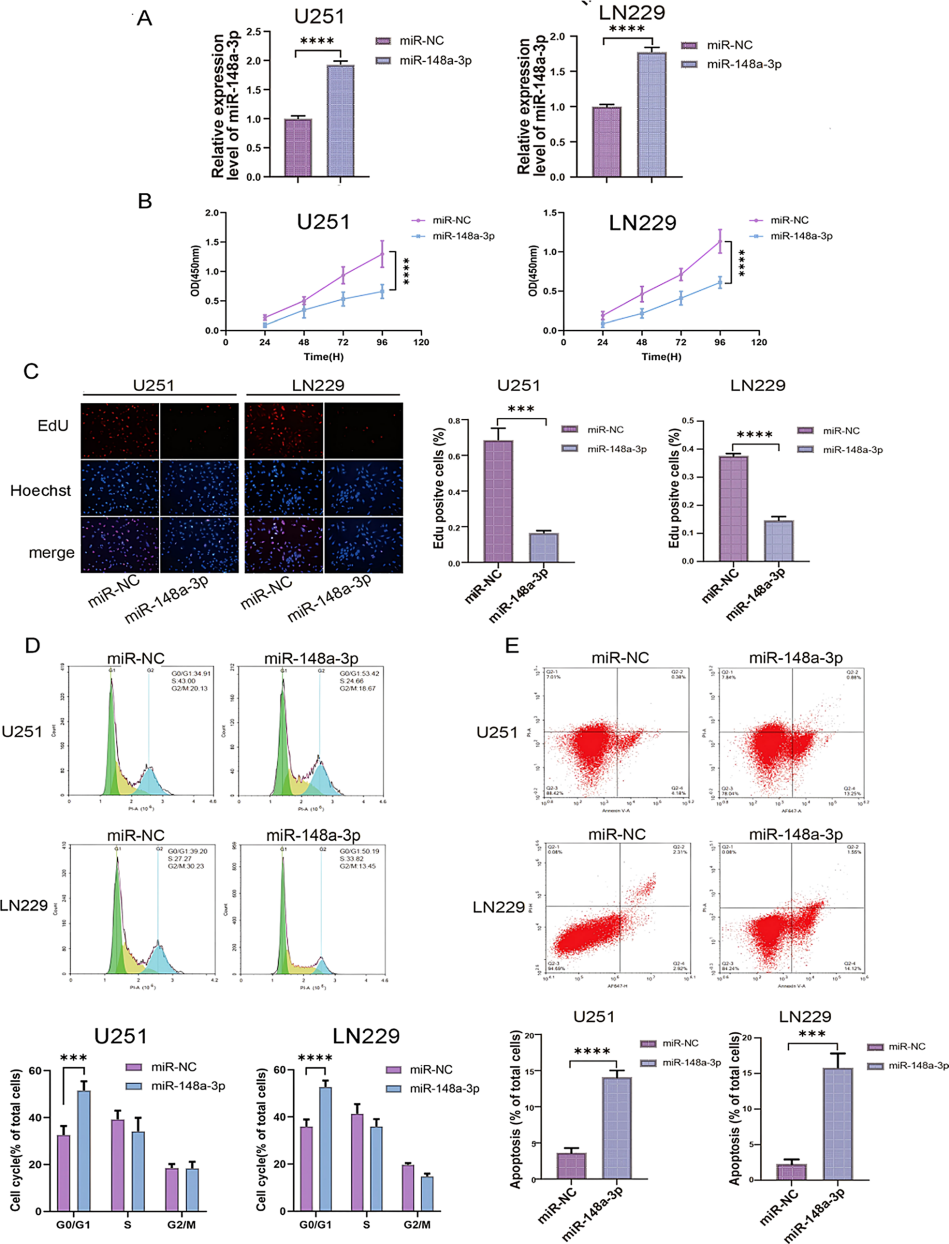
miR-148a-3p inhibits glioma cell proliferation and cell cycle progression, while promoting glioma cell apoptosis. Functional assays assessed cells viability, proliferation, cell cycle, and apoptosis using CCK-8 (**A**), EdU (**B**), and flow cytometry (**C**, **D**). Data represent three independent experiments. Statistical significance was determined by Student’s t-test (two groups) and one-way ANOVA test and Tukey’s post-hoc test (multiple groups), with *p* < 0.05 considered significant (*p < 0.05, **p < 0.01, ***p < 0.001, ****p < 0.0001)

Once we verified that SIRT7 is truly a downstream target of miR-148a-3p, our next move was to explore whether miR-148a-3p exerts functional effects on glioblastoma cell behavior through this regulatory association. Following this, the growth rate of cells transfected with miR-148a-3p mimics was significantly reduced when compared to that of cells transfected with miR - NC (Fig. 5A - B). Flow cytometry examination indicated that cells transfected with miR-148a-3p were arrested in the G1 phase (Fig. 5C), and the number of apoptotic cells increased. These results were consistent with those derived from cells transfected with the shSIRT7 lentivirus (Fig. 5D).

### SIRT7 overexpression can reverse the role of miR-148a-3p overexpression on proliferation, cell cycle progression, and apoptosis of glioblastoma cells

**Fig. 6:**
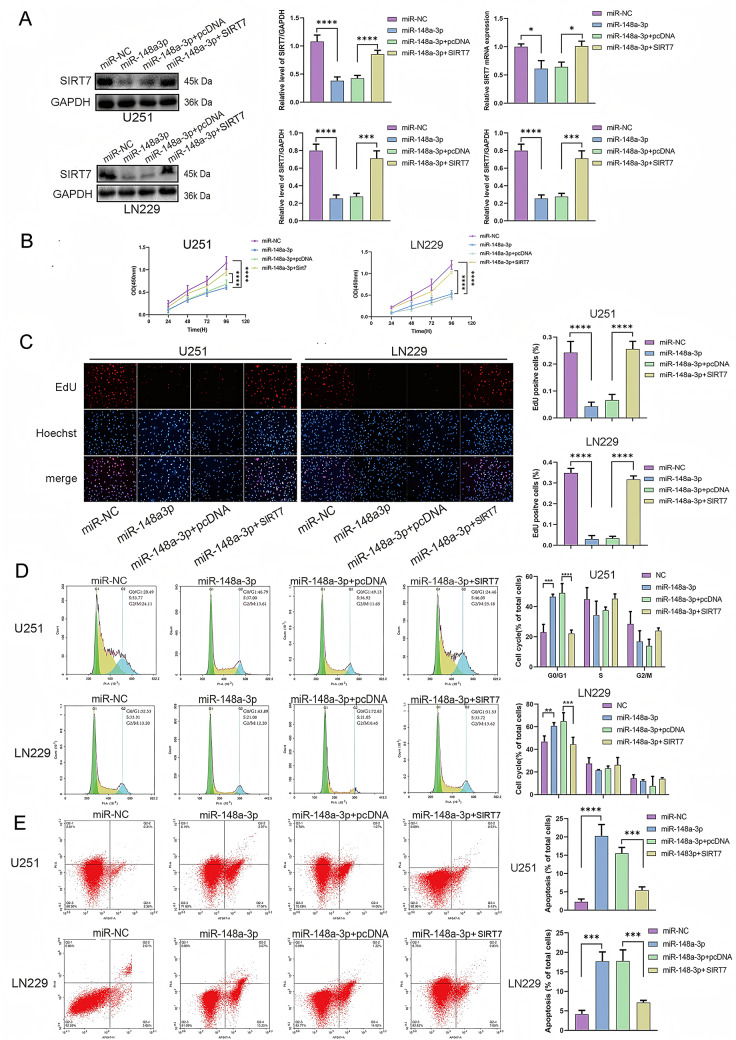
The overexpression of SIRT7 counteracts the inhibitory impacts of miR-148a-3p in glioma cells. (**A**) The expression levels of SIRT7 mRNA and protein in transfected glioma cells were determined via qRT - PCR and western blot analysis. The experimental groups included the miR-148a-3p negative control group (miR - NC), the miR-148a-3p overexpression group (miR-148a-3p mimics), the SIRT7 overexpression group (cDNASIRT7), and the SIRT7 overexpression control group (pcDNA). (**B**,** C**) Cell viability was assessed using the CCK − 8 assay and the EdU assay (Magnification: 10×). (**D**) The cell cycle was analyzed by flow cytometry. (**E**) Cell apoptosis was assessed by flow cytometry. Data are from three independent experiments. Statistical analysis used Student’s t-test (two groups) and Tukey’s post-hoc test (multiple groups), with *p* < 0.05 considered significant (*p < 0.05, **p < 0.01, ***p < 0.001, ****p < 0.0001)

Since miR-148a-3p inhibited the proliferation of glioma and induced apoptosis in a manner comparable to the consequences of SIRT7 knockdown, rescue experiments were carried out to ascertain whether these effects were specifically mediated through SIRT7. Assays confirmed that SIRT7 overexpression could counteract the effect of miR-148a-3p mimics on SIRT7 expression in U251 and LN229 cells (Fig. 6A). After infecting miR-148a-3p-treated U251 and LN229 cells with cDNA SIRT7 lentivirus, the levels of proliferation, cell cycle progression, and apoptosis were altered (Fig. 6B-E). The findings gathered from this research offered considerable proof in favor of the hypothesis that miR-148a-3p has a regulatory impact on the progression of glioblastoma cells by specifically zeroing in on SIRT7.

### SIRT7 knockdown enhanced the cytotoxicity of TMZ in glioblastoma cells

**Fig. 7:**
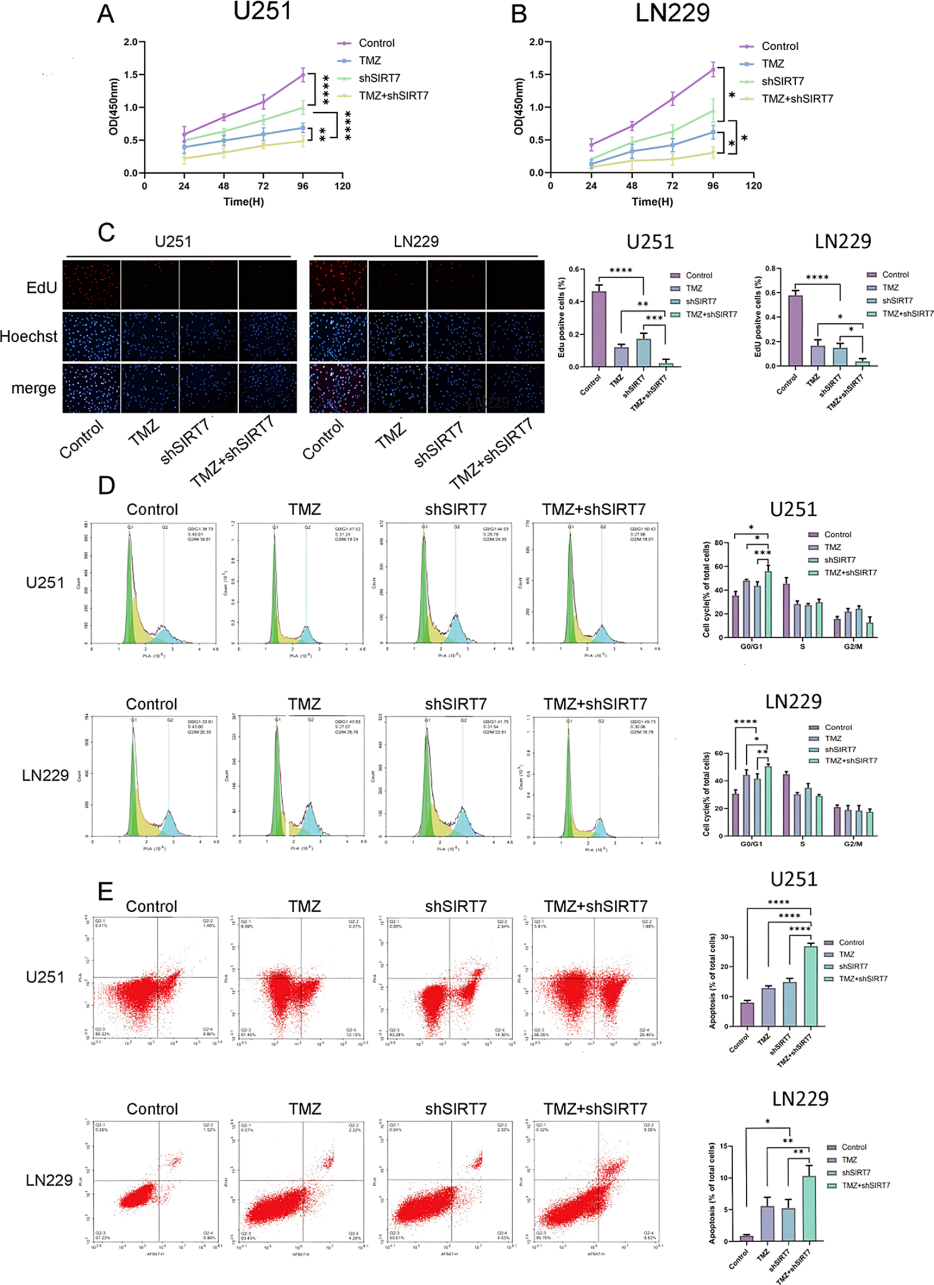
Effect of SIRT7 downregulation on TMZ-induced cytotoxicity in glioma cells. (**A-C**) U251 and LN229 cells were divided into 4 groups and treated them with (1) physiological saline (control), (2) TMZ, (3) shSIRT7, or (4) TMZ + shSIRT7 and cell viability of the above groups of glioma cells were detected by CCK-8 assay and EdU assay (Magnifcation: 10×). (**D**) Cell cycle was estimated by flow cytometry. (**E**) Cell apoptosis was measured by flow cytometry. Data are from three independent experiments. Statistical analysis used Student’s t-test (two groups) and Tukey’s post-hoc test (multiple groups), with *p* < 0.05 considered significant (*p < 0.05, **p < 0.01, ***p < 0.001, ****p < 0.0001)

As SIRT7 is a validated downstream target of miR-148a-3p and promotes glioblastoma cell proliferation and survival, we next investigated whether its suppression could enhance the efficacy of TMZ, a standard chemotherapeutic agent used in glioma treatment. We divided U251 and LN229 cells into four groups and treated them with (1) physiological saline (control), (2) TMZ, (3) shSIRT7 lentivirus, or (4) TMZ + shSIRT7 lentivirus. The findings indicated that in U251 and LN229 cells exposed to temozolomide (TMZ), there was a notable decrease in cell proliferation (Fig. 7A − 7 C). Moreover, the proportion of cells undergoing apoptosis was higher compared to that in the control cells treated with physiological saline (Fig. 7D). An examination of the cell cycle demonstrated a substantial build - up of cells in the G1 phase (Fig. 7E). When the expression of SIRT7 was suppressed, the cytotoxic impact of temozolomide (TMZ) on LN229 and U251 cells was significantly increased. Specifically, there was a decrease in the cell proliferation rate (Fig. 7A − 7 C). Meanwhile, the degree of apoptosis went up (Fig. 7D), and a greater number of cells were halted in the G1 phase (Fig. 7E). The findings imply that the inhibition of SIRT7 augmented the cytotoxic potential of temozolomide (TMZ) in glioblastoma cells.

### Downregulation of SIRT7 inhibited glioma tiuuses enlargement and heightened antitumor effects mediated by TMZ in U251 cell-derived glioma xenotransplantation mouse models

**Fig. 8:**
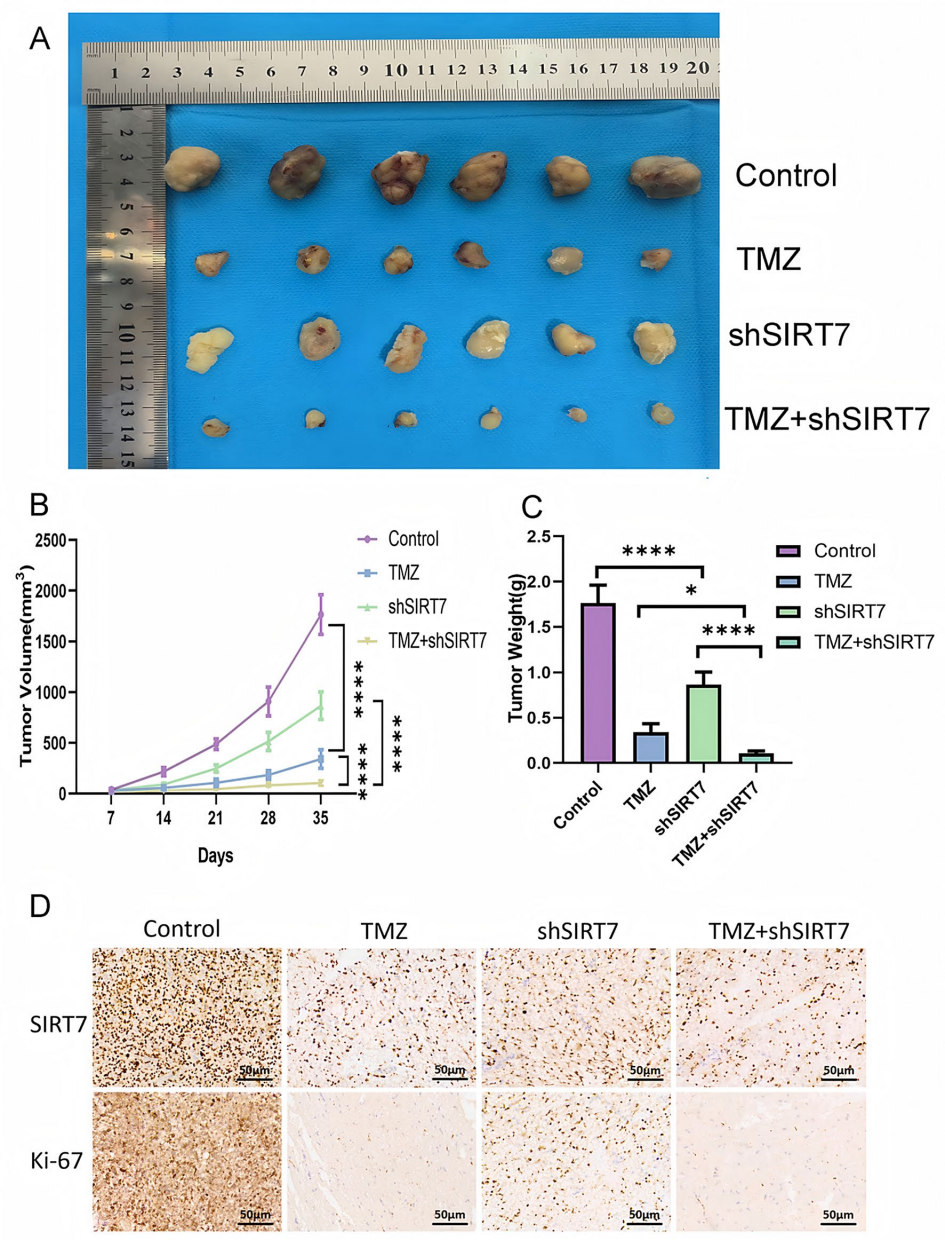
In vivo, the suppression of SIRT7 makes glioma xenografts more susceptible to treatment with TMZ. (**A**) Visual representations of xenograft tumors were obtained from four sets of BALB/c - nude mice. (**B**) The size of the tumors was gauged every seven days using a caliper. (**C**) Upon completion of the experiments, the tumors were removed and their weights were determined. (**D**) Immunohistochemical analysis was carried out on tumor sections to detect SIRT7 and Ki − 67. The data presented in (**A - D**) were derived from a single experiment involving six mice in each group. Statistical analysis used Student’s t-test (two groups) and Tukey’s post-hoc test (multiple groups), with *p* < 0.05 considered significant (*p* < 0.05; ***p < 0.0001)

To validate these in - vivo findings and investigate the possible therapeutic uses of targeting SIRT7, we developed glioma xenograft models in nude mice. Subsequently, we evaluated the tumor’s responses to temozolomide (TMZ) by comparing the scenarios in which SIRT7 was silenced with those in which it was not. As expected, tumor volumes were lower in both of the TMZ-treated model groups than those in the non-TMZ treated groups, but the lowest values were observed for the TMZ + shSIRT7 group (Fig. 8A-C). In addition, immunohistochemical staining results of mouse glioma tissues provided strong evidence that SIRT7 knockdown enhanced TMZ-induced cytotoxicity in glioblastoma cells (Fig. 8D).

## Discussion

Our research results confirm that the miR-148a-3p/SIRT7 axis serves as a crucial modulator of glioma aggressiveness and the sensitivity of glioma cells to temozolomide (TMZ). In particular, we determined that SIRT7 is a verified downstream target of miR-148a-3p. Moreover, we demonstrated that the inhibition of SIRT7, achieved either through miRNA overexpression or shRNA - mediated down - regulation, restricted the proliferation of glioblastoma cells. It triggered cell cycle arrest and apoptosis, and augmented the cytotoxic effects of TMZ both in the in - vitro setting and in in - vivo organisms. These findings offer novel perspectives on the mechanisms of molecular regulation of glioma malignancy and chemotherapy resistance.

As disease research has shifted from the cellular level to the molecular level, molecular biomarkers not only improves diagnostic accuracy and prognosis prediction but also helps improve our understanding of disease development and progression [20]. Sirtuin family proteins have received much attention from researchers owing to their role in numerous physiological and pathological processes [21], such as the cell cycle [22], mitochondrial homeostasis [23], autophagy [24], cell growth [25], and protein activity and stability [26]. Most previous studies on sirtuin family proteins focused on their regulatory role in aging [27–29]. However, increasing evidence has shown that the Sirtuins family plays important roles in metabolic regulation [30, 31], stress response [32], inflammatory response [33], and tumor development [34]. SIRT7, the most recently identified Sirtuin, is still poorly characterized. With its special localization in the nucleolus, SIRT7 participates in cell metabolism [35], cell stress [36], and DNA damage repair [37], through various mechanisms. SIRT7 plays crucial regulatory roles in the development of various cancers, for example, head and neck squamous cell carcinoma [37]. and liver [38], gastric [39], breast [40], bladder [41], colorectal [42], and pancreatic [43] cancers. However, its role in glioma has not been fully explored. Our data showed that SIRT7 is obviously expressed in glioma tissues and correlates with higher tumor grade and poorer prognosis, consistent with its oncogenic role. Functionally, both gain- and loss-of-function assays confirmed that SIRT7 is essential for maintaining glioblastoma cell growth and survival.

Notably, we identified a new upstream regulatory mechanism for SIRT7 by determining miR-148a-3p as a direct post - transcriptional suppressor. Recently, miR-148a-3p has emerged as an anti - cancer miRNA in various cancer types, including glioma. Zhang and co - workers demonstrated that miR-148a-3p affects the progression of hepatocellular carcinoma via the ITGA5/PI3K/Akt pathway [44]. Ouyang and colleagues discovered that miR-148a-3p inhibited the mobility of colon cancer cells by zeroing in on HDAC5 [15]. Liang and the research group disclosed that miR-148a-3p hindered the advancement of lung adenocarcinoma by targeting MAP3K9 [15]. In a study that compared the miRNA profiles in tumors of long - term and short - term surviving glioblastoma patients, miR-148a-3p exhibited a significant disparity between the two groups and was upregulated in long - term survivors [19]. Another study suggested that miR-148a-3p was associated with necrotic apoptosis in tumor cells [45]. In our study, the expression of miR-148a-3p was significantly reduced in glioblastoma cells in comparison to NHA cells. When the expression of miR-148a-3p was restored, it mimicked the outcomes of SIRT7 knockdown. Subsequently, re - expressing SIRT7 reversed these outcomes. This finding affirmed the functional importance of this regulatory axis in glioma.

Intriguingly, we observed that the expression of miR-148a-3p in the U87MG cell line appears statistically indistinguishable from that in the other two lines, whereas downstream SIRT7 expression differs, suggesting that SIRT7 is not solely regulated by miR-148a-3p. Literature searches revealed the following explanation: although miR-148a-3p levels do not differ significantly between U87MG and other cell lines, SIRT7 remains highly expressed, implying that additional miRNAs, RNA-binding proteins, or epigenetic mechanisms concurrently modulate SIRT7. This observation aligns with the current view of SIRT7 as a node subjected to multi-layered regulation, and future work will explore whether mRNA stability or 3’-UTR variants contribute to this phenotype.

Our investigation additionally presents convincing evidence that focusing on the miR-148a-3p/SIRT7 axis enhances the treatment efficacy of TMZ. Although TMZ remains the standard treatment for glioma, its efficacy is often compromised by intrinsic or acquired resistance. Notably, SIRT7 knockdown sensitized glioblastoma cells to TMZ-induced apoptosis and growth inhibition, and this effect was confirmed by subcutaneous tumorigenesis in nude mouse. These results suggest that SIRT7 may be involved in TMZ resistance through its anti-apoptotic and pro-survival functions, and that regulation of the miR-148a-3p/SIRT7 axis may provide a promising strategy to overcome chemotherapy resistance in glioma.

Notwithstanding these discoveries, our research confronts a number of constraints. Firstly, even though we verified that miR-148a-3p directly acts on SIRT7, we cannot rule out the participation of other targets or downstream pathways that could possibly account for the observed phenotypes. Second, while our in vivo experiments support the therapeutic potential of SIRT7 inhibition, further studies using orthotopic glioma models and clinical-grade delivery systems with miRNA mimics or inhibitors are needed to verify the applicability of translation. Finally, the molecular mechanisms by which SIRT7 regulates TMZ response, such as DNA repair pathways or apoptotic signaling pathways, warrant further investigation.

Overall, our discoveries identify the miR-148a-3p/SIRT7 axis as a vital mechanistic controller that manages both the progression of glioma and the sensitivity to chemotherapy with temozolomide. This mechanistic proof expands our knowledge of glioma biology. Additionally, it implies that re - establishing the expression of miR-148a-3p or directly targeting SIRT7 could be a new therapeutic approach to enhance the prognosis of patients with glioma.

## Conclusions and limitations

Collectively, our findings establish the miR-148a-3p/SIRT7 axis as a central mechanistic regulator governing both glioma progression and temozolomide chemosensitivity. This mechanistic evidence expands our understanding of glioma biology and suggest that restoring miR-148a-3p expression or directly targeting SIRT7 may serve as a novel therapeutic strategy for improving outcomes in glioma patients. Despite these findings, our study has several limitations. First, although we confirmed that miR-148a-3p directly targets SIRT7, we cannot exclude the involvement of other targets or downstream pathways that may contribute to the observed phenotypes. Second, while our in vivo experiments support the therapeutic potential of SIRT7 inhibition, further studies using orthotopic glioma models and clinical-grade delivery systems with miRNA mimics or inhibitors are needed to verify the applicability of translation, and then the downstream molecular mechanisms of SIRT7 that mediate the pro-tumorigenic and TMZ-sensitizing effects remain to be elucidated and are one of our ongoing study. Last but no least, as a research tool possessing the unique ability to recapitulate the cellular hierarchy, invasive behavior, and therapeutic resistance observed in human gliomas, tumor stem cells were not integrated into our study to improve the translational relevance of our findings.

## Data Availability

The data that support the findings of this study are openly available in The Cancer Genome Atlas Program (TCGA) at https://www.cancer.gov/ccg/research/genome-sequencing/tcga, reference number, and Chinese Glioma Genome Atlas (CGGA) at http://www.cgga.org.cn/.
